# The Impact of Cognitive‐Behavioral Therapy on Suicidal Ideation and Despair Among Individuals With a History of Suicide Attempts: A Randomized Controlled Trial

**DOI:** 10.1002/hsr2.71187

**Published:** 2025-08-19

**Authors:** Zahra Kermanshahi, Leyla Alilu, Moulad Radfar, Behzad Bushehri, Hamidreza Khalkhali

**Affiliations:** ^1^ Department of Psychiatric Nursing, School of Nursing and Midwifery Urmia University of Medical Sciences Urmia Iran; ^2^ School of Nursing and Midwifery Urmia University of Medical Sciences Urmia Iran; ^3^ School of Medicine Urmia University of Medical Sciences Urmia Iran; ^4^ Biostatistics and Epidemiology Department, School of Medicine Urmia University of Medical Sciences Urmia Iran

**Keywords:** cognitive‐behavioral therapy, despair, suicidal ideation, suicide

## Abstract

**Background and Aims:**

Suicide and suicide attempts are critical indicators of psychological well‐being within societies, influenced by various factors. Cognitive‐behavioral therapy represents a prominent intervention for individuals experiencing suicidal ideation and despair, offering potential alleviation of depressive symptoms. Consequently, this study aimed to assess the effects of cognitive‐behavioral therapy on suicidal ideation and despair among individuals with a history of suicide attempts.

**Methods:**

This single‐blind experimental study was conducted in 2022 in Iran. In this study, a total of 60 individuals with a history of recent suicide attempts who were discharged from teaching hospitals affiliated with Urmia University of Medical Sciences were selected using convenience sampling and assigned to two intervention (30 people) and control (30 people) groups. Data were collected at three time points before, 1 month after, and 3 months after the intervention using a demographic questionnaire, Beck Suicidality Scale (BSSI), and Beck Hopelessness Questionnaire (BHS). The intervention group received eight sessions of cognitive behavioral therapy, while the control group did not receive any intervention.

**Results:**

Analysis of variance revealed a significant distinction in mean scores of suicidal ideation and despair between the intervention and control groups (*p* < 0.001). Pairwise comparisons based on the Bonferroni test also showed that there was a significant difference between the mean baseline score of suicidal ideation and hopelessness and the mean scores after the intervention, 1 month, and 3 months after the intervention (*p* < 0.001). However, this difference was not significant in the control group (*p* = 1.000).

**Conclusion:**

The study demonstrated the efficacy of cognitive‐behavioral therapy within the intervention group, resulting in notable improvements in suicidal ideation and despair symptoms. Consequently, cognitive‐behavioral therapy, through mechanisms such as acceptance, mindfulness, present‐focused awareness, non‐judgmental observation, and avoidance of cognitive distortions, holds promise in reducing suicidal ideation and despair among individuals with a history of suicide attempts.

## 1 Introduction

Suicide and attempted suicide are considered to be among the most important indicators of the psychosocial health of individuals in a society. Suicide usually manifests itself in the form of death by suicide, attempted suicide, and suicidal thoughts. Died by suicide includes all cases in which a person causes harm to themselves by performing a destructive act that leads to death. Attempted suicide consists of those actions that a person takes to destroy himself, but does not lead to death [1]. The term suicidal thoughts refers to mental preoccupations that range from fleeting thoughts about the worthlessness of life and the desire to die to practical plans for self‐destruction [2]. Therefore, suicidal thoughts themselves are considered a risk factor for suicide [3].

Suicide rates have been on the rise in recent years, which has not only increased tension and concern among communities but has also affected the world [4]. The World Health Organization and the International Association for Suicide Prevention designated September 10 as World Suicide Prevention Day because of this alarming increase [5]. In the United States, about 6.4% of adults attempt suicide, and 14% of them have serious suicidal thoughts [6]. A study of 171 students showed that approximately one‐third of them had suicidal thoughts during their lifetime [7].

The first World Health Organization psychotherapy program was approved at the 66th World Health Assembly in May 2013. Suicide prevention was an integral part of the plan, which aimed to reduce the country's suicide rate by 10% by 2020. The average annual global suicide rate is 11.4 per 100,000 population (15 in men and 8 in women). However, because suicide is a sensitive issue and is even illegal in some countries, it is likely underreported [8].

In Iran, according to the country's Forensic Medicine Organization, the total number of suicide deaths in 2017 was 4,627, compared to 2016, a 5% increase. 3,262 men and 1,365 women were reported to have died by suicide, and yet the suicide rate in Iran is lower than in the world; this figure in Iran is about five per 100,000 people, lower than in Western countries but higher than in other Middle Eastern countries [9]. In a systematic review and meta‐analysis conducted on data from 16 provinces with 12,005 samples in Iran, Nazarzadeh and colleagues found that only eight studies reported a mean age of 21.9 years [10].

Based on the suicide readiness model, researchers emphasized the relationship between negative life stressors, cognitive distortion, poor problem‐solving skills, and hopelessness in developing suicidal thoughts or attempting suicide. According to this model, a person who is deficient in divergent thinking is cognitively unprepared to deal with high levels of psychological stress in life and is likely to become frustrated under such conditions [11]. Despair is defined as the expectation of the nonoccurrence of positive events or the expectation of adverse events [12]. Melges and Bowlby consider the reduction in expectation of success to be the main component of despair. In their view, a person will become discouraged when his or her current action plans no longer seem capable of paving the way to achieving his or her long‐term goals, but at the same time, the person is unable to ignore his or her past goals [13]. A pessimistic outlook on the future can serve as a significant indicator of recurrent suicidal behavior and is linked to an increased risk of eventual suicide. There is evidence that in individuals at risk of suicide, greater hopelessness leads to fewer positive events and more negative events being anticipated [14].

Since suicide causes numerous physical, psychological, and social complications, including disability and family dissolution, abandoning a person who has died by suicide will not only increase the likelihood of suicide recurrence but will also mean the continuation of the individual's and family's abnormalities [15]. The diversity and multiplicity of causes of suicide indicate that suicide prevention requires a multi‐faceted approach with special attention to mental health [16]. Psychotherapy is a type of social interaction in which a professional helps a patient change their behaviors and feelings. One type of psychotherapy is cognitive therapy, and the basis of this method is based on current issues and problems and their resolution. This method is used both individually and in groups. Cognitive therapy, by creating changes and transformations in the individual's cognitive system, cognitive therapy causes the individual's reactions to be qualitatively changed and the person can correctly understand and change realities [17].

Cognitive‐behavioral therapy (CBT), a derivative of cognitive therapy, is a psychotherapeutic approach that addresses maladaptive patterns through the modification of thoughts and behaviors. It operates on the principle of interconnectedness among cognition, affect, somatic states, and behavioral outcomes, facilitating comprehensive problem management [18]. Because thoughts and actions often overlap, behavioral and cognitive approaches are often used with the same patient, and the term cognitive behavioral therapy (CBT) has become popular to emphasize their close relationship [19]. Cognitive behavioral therapy is a process that enables patients to recognize their false beliefs and misconceptions that lead to low mood, negative behaviors, and low self‐esteem [20]. In this way, the client initially accepts some negative perceptions and interpretations about herself that lead to the emergence of negative thoughts in the individual, and then, in the next stage, learns to recognize negative thoughts in herself and discover and replace them with new and healthy thoughts that are closer to reality [21].

According to a study by Kiani et al. (2010), cognitive‐behavioral group therapy can reduce hopelessness and increase self‐esteem at the general, family, and educational levels [22]. A study by Mohyadini et al. (2015) The findings demonstrated that group‐based cognitive‐behavioral therapy significantly reduced feelings of hopelessness among female participants with suicidal ideation [23]. In 2016, Moharrami et al. showed that, given that hopelessness is a predictor of suicide and a key symptom of depression, cognitive‐behavioral therapy based on Beck's model is effective and beneficial. This treatment is also effective in reducing the symptoms of apathy in depressed patients, but not as much as hopelessness [24]. Jafari et al. (2014) found comparable efficacy between mindfulness‐based cognitive therapy and behavioral activation in improving quality of life and reducing suicidal ideation among depressed individuals [25]. Also, Dimidjian et al. (2009) placed great emphasis on correcting patients' ineffective thoughts with this method [26].

Since experiencing suicidal ideation increases the likelihood of suicide attempts and death by suicide events, identifying the cognitive processes that lead to suicidal ideation can be effective in screening, preventing, assessing, and treating suicide. Given the scarcity of studies on cognitive‐behavioral therapy's impact on Suicidal Ideation and Despair among individuals with a history of suicide attempts, particularly within the Iranian context, the present research is designed to examine the efficacy of cognitive‐behavioral therapy on suicidal Ideation and Despair within Urmia's educational and medical centers in 2022.

## 2 Materials and Methods

### 2.1 Study Design and Setting

This study was a single‐blind RCT conducted in Urmia, Iran, at two major medical centers: Taleghani and Imam Khomeini Hospitals, during the year 2022.

### 2.2 Participants and Sample Size

The required sample size was determined using data from Mohyadini et al. (2015), which reported mean scores (±SD) of 11.62 ± 3.49 (intervention group) and 15.49 ± 3.27 (control group). With a 95% confidence interval and test power, the minimum sample size per group was calculated as 24 using the specified formula:

$$n=\frac{{\left(Z_{\frac{\alpha }{2}}+Z_{\beta }\right)}^{2}(S_{1}^{2}+S_{2}^{2})}{{\left({\overset{®}{x}}_{1}-{\overset{®}{x}}_{2}\right)}^{2}}=n1=n2=\frac{{\left(1/96+1/96\right)}^{2}({3/49}^{2}+{3/27}^{2})}{{\left(11/62-15/49\right)}^{2}}≅24$$

To account for a potential 20% attrition rate, the final sample size was adjusted to 30 participants per group, resulting in a total of 60 participants across both groups.

### 2.3 Inclusion and Exclusion Criteria

The inclusion criteria included the following: (a) history of at least one suicide attempt, (b) age 18 years or older, (c) absence of drug addiction and bipolar disorder, (d) voluntary consent for study participation, (e) absence of physical impairments, (f) no previous involvement in similar research studies, (g) capacity to engage in cognitive‐behavioral therapy sessions, (h) ability to communicate and respond to inquiries effectively. The exclusion criteria consisted of (a) participant reluctance to continue cooperation and (b) missing more than two therapy sessions.

### 2.4 Data Collection

Data collection was performed using a demographic questionnaire, the Beck Scale for Suicidal Ideation (BSSI), and the Beck Despair Questionnaire (BHS).

**The demographic questionnaire** comprised questions regarding participants' age, gender, marital status, income level, education, employment status, history of physical illnesses, suicide occurrences, timing, method, and causes of suicide.

**The beck despair questionnaire (BHS)** was developed by Aaron T. Beck in 1979. This tool consists of 20 items requiring individuals to respond with “correct” or “incorrect” based on each statement. This questionnaire, which targets individuals aged 17 to 80, assesses three facets of despair: attitudes toward the future, motivation levels, and expectations. The total possible score for this instrument spans from 0 to 20. The higher scores correlate to increased levels of despair [27].

Beck and Steer's 1988 investigation reported internal consistency coefficients of 0.92 and 0.93 for the Beck Despair Scale among individuals with suicidal ideation and suicide attempters, respectively [28]. Durham further affirmed the reliability of this scale, yielding a coefficient of 0.86 among psychiatric populations [29]. In an Iranian study, the Beck Despair Questionnaire exhibited a Cronbach's alpha of 0.87 for the overall scale and between 0.83 and 0.86 for the subscales [30].

**The Beck Scale for Suicidal Ideation (BSSI)**, developed by Aaron Beck in 1961, serves to assess suicidal ideation intensity and gauge attitudes, behaviors, and plans related to suicide over the preceding week. This tool comprises a 19‐item self‐assessment instrument, with each query offering three response options scored via the Likert method from zero to 2. Total scores on this scale range from 0 to 38, with interpretations indicating the presence of suicidal ideation (0–5), inclination towards suicide [6, 7, 8, 9, 10, 11, 12, 13, 14, 15, 16, 17, 18, 19], or intent to commit suicide [20, 21, 22, 23, 24, 25, 26, 27, 28, 29, 30, 31, 32, 33, 34, 35, 36, 37, 38]. According to Beck and Steer's research, the reliability of the Beck Scale for Suicidal Ideation, assessed using the Cronbach's alpha method, ranges from 0.87 to 0.97. In contrast, the test‐retest method yields a reliability coefficient of 0.54 [31]. Anisi et al.'s 2005 study evaluated the validity and reliability of the Beck Suicidal Thought Scale among 133 males aged 19 to 29, selected through convenience sampling. The scale demonstrated a reliability of 95% using Cronbach's alpha and 88% with the two halves method, indicating acceptable reliability. The findings underscore the Beck Scale for Suicidal Ideation's efficacy in detecting suicidal thoughts, aligning with suicide definitions, and serving as a valid and reliable assessment tool [32].

### 2.5 Ethical Considerations

All stages of this study involving human participants adhered to the principles outlined in the Declaration of Helsinki. The study protocol was approved by the Ethics Committee of Urmia University of Medical Sciences, Urmia, Iran (IR. UMSU. REC.1399.321), and the trial was registered in the Iranian Registry of Clinical Trials before sampling (Registration No. IRCT20161116030926N8). All participants provided informed written consent after being thoroughly informed about the study's objectives and procedures. Participant anonymity was maintained by coding the questionnaires.

### 2.6 Intervention

Following the approval from the administrative authorities of Imam Khomeini and Taleghani Hospitals, the sampling process was initiated. Sixty eligible individuals meeting the inclusion criteria were chosen through convenience sampling upon their discharge from the hospital following suicide attempts within 3 days but no longer than 3 weeks after the suicide attempt, depending on individual circumstances and patient readiness. To assign participants to either the intervention or control group, 30 cards labeled “A” (intervention) and 30 cards labeled “B” (control) were placed in sealed envelopes. The researcher acquainted the eligible individuals with the research objectives and secured their informed consent. Participants were asked to randomly select an envelope, determining their placement in either the control or intervention group based on the cards. As individuals were selected incrementally, group formation commenced once eight participants were gathered.

Before the intervention, both control and intervention groups completed demographic surveys, the Beck Suicidal Thought Scale, and the Beck Despair Questionnaire. Ensuring anonymity, participants were assured of the confidentiality of their responses, with data solely used for research objectives, and the option for participants to receive study results.

An educational program was developed for the intervention group, followed by cognitive‐behavioral therapy conducted at the Imam Khomeini Hospital amphitheater across eight sessions lasting 60–90 min each, held twice weekly by the first researcher, a master's student in psychiatric nursing under the guidance of a psychiatric nursing consultant (third researcher) and a forensic attending physician (fourth researcher). All cognitive therapy sessions were monitored by the supervisor, and the therapist was given the necessary feedback. A list of cognitive‐behavioral therapy steps and sections was also prepared, and the supervisor checked whether all steps were implemented and provided the necessary guidance.

Participants missing up to two sessions received individual catch‐up sessions; however, those absent for more than two sessions were excluded from the study. No interventions were administered to the control group during the study period, but post‐study, the cognitive‐behavioral therapy content was shared with them through an educational booklet. Cognitive‐behavioral therapy issues are described in Table 1. Participants in both study groups received treatment as usual (Psychiatric outpatient visits for treatment and counseling), but unfortunately, there was no facility to receive routine care from community physicians, as well as follow‐up and referral services from case managers in this study.

**Table 1:** Content of the sessions of cognitive‐behavioral therapy.

Session no	Content
**Session 1**	‐ Introducing the group therapist and getting to know each other, reviewing the goals of forming the group, reviewing the structure of the sessions and the main rules ‐ Obtaining a written commitment from group members not to attempt suicide until the end of the intervention sessions ‐ Discussing and exchanging information about suicidal thoughts and factors affecting them ‐ Conclusion
**Session 2**	‐ Collecting information about the individual and family's situation and encouraging family members to tell their life stories, creating empathy, facilitating the tools of fear ‐ Gaining awareness of the communication pattern and painful experiences of members ‐ Teaching communication skills and listening skills and good conversation strategies ‐ Conclusion and presenting homework and discussing its importance in the therapeutic process (reviewing conversations with those around them and paying attention to how often the barriers to listening are used)
**Session 3**	‐ Reviewing homework ‐ Introducing the ABC technique with relevant examples (understanding that the type of interpretation of events causes committed behavior) ‐ Identifying types of stimuli or cognitive errors and negative and ineffective automatic thoughts ‐ Receiving feedback and presenting homework (writing 3 of the worst life events based on the ABC model and 3 of the events that showed a different way of thinking with loved ones)
**Session 4**	‐ Reviewing members' homework ‐ Continuing to identify distortions or cognitive errors ‐ Members talking about their thoughts and beliefs ‐ Identifying members' negative views of themselves and the future ‐ Teaching challenges with cognitive distortions and providing homework (practicing clear thinking and providing questions to group members to evaluate, clarify, and reconstruct thoughts)
**Session 5**	‐ Reviewing the assignments from the previous session ‐ Implementing the Downward Arrow Technique (Identifying central beliefs or beliefs that are the core of the patient's problems and changing them and replacing negative thoughts with effective and realistic thoughts) ‐ Implementing the Confirmatory Evidence and Rebuttal Evidence Technique so that the patient can gain insight into their dysfunctional beliefs and realize the healthy and positive aspects of their capabilities in life). ‐ Strengthening strengths and positive feelings and emotions ‐ Summarizing and presenting homework (practicing and repeating techniques)
**Session 6**	‐ Reviewing the assignments from the previous session ‐ Teaching the flashcard technique for long‐term effectiveness of the technique of confirming and refuting evidence and to help remember, combat inappropriate cognitions, and help achieve positive and appropriate behavior, especially when the person is struggling with suicidal beliefs. ‐ Practicing the flashcard technique and explaining its importance to group members ‐ Teaching problem‐solving skills, examining the benefits and drawbacks and obstacles of each solution, and providing compensation solutions to overcome obstacles ‐ Summarizing and presenting homework (practicing and repeating the flashcard technique and preparing several flashcards and practicing problem‐solving skills and evaluating their results)
**Session 7**	‐ Reviewing members' homework ‐ Presenting sleep hygiene techniques (most people who are depressed or whose minds are full of negative and self‐harming thoughts have problems in terms of quality and quantity of sleep) ‐ Teaching methods for achieving relaxation, including relaxation techniques and implementing them step by step and practicing with members ‐ Teaching and practicing diaphragmatic breathing ‐ Summarizing and presenting homework (practicing sleep hygiene and relaxation techniques and evaluating and reporting their results)
**Session 8**	‐ Reviewing homework ‐ Providing a summary of the sessions ‐ Teaching the attention‐redirection technique (this technique is involved in regulating and controlling attention and helps group members to shift their attention from internal cues (the situation emotional and negative automatic thoughts) and external cues such as the urge to harm oneself. ‐ Encourage group members to write down daily situations that lead to thought temptation and implement attention redirection techniques ‐ Implement a relapse prevention program (identify factors that will cause relapse and ways to deal with them

### 2.7 Data Analysis

All statistical analyses were performed using IBM SPSS Statistics for Windows, Version 22.0 (IBM Corp.). The Kolmogorov–Smirnov test was used to evaluate the normality of data distribution. Descriptive statistics were reported as frequencies and percentages for categorical variables, and as means with standard deviations for continuous variables with normal distribution. For inferential analysis, the *χ*² test was applied to evaluate group homogeneity, and repeated measures analysis of variance (rANOVA) was used for within‐group comparisons. A *p*‐value of less than 0.05 was considered statistically significant for all analyses. Additionally, data analysis was performed by an independent researcher who was blinded to group assignments.

## 3 Results

**Figure 1:**
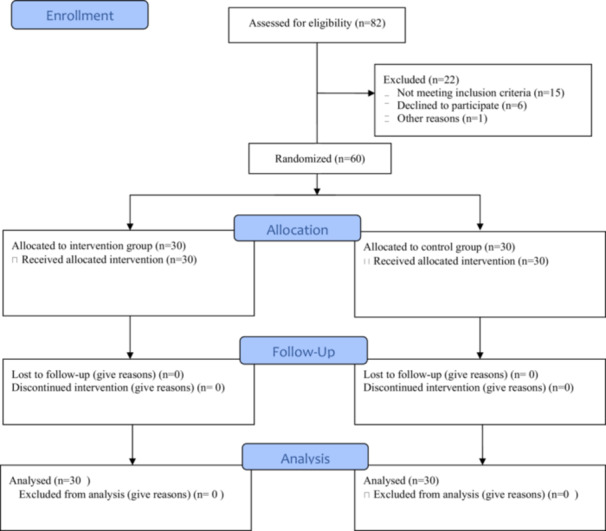
Flow diagram of the study selection process.

Out of 82 participants assessed, 15 were excluded due to ineligibility, six due to unwillingness to participate (before randomization and completing the pre‐test), and one due to other reasons (before randomization and completing the pre‐test). Subsequently, 30 participants were randomly allocated to the intervention group and 30 to the control group (Figure 1).

### 3.1 Demographic Characteristics

According to the results of this study, the mean age of participants in the control group was 29.26 ± 9.07 years, while the mean age in the intervention group was 27.73 ± 7.23 years. The results of the independent *t*‐test indicated no statistically significant difference in age between the intervention and control groups (*t* = 0.724, *p* = 0.472). The results of the *χ*² test indicated no statistically significant differences between the intervention and control groups in terms of gender, marital status, or income level, education level, occupation, history of physical illness, suicide occurrences, timing, method, and causes of suicide (*p* > 0.05). In other words, the two groups were demographically comparable, with no significant differences observed in baseline characteristics (Table 2).

**Table 2:** Comparison of quantitative and qualitative demographic characteristics between the two groups.

Qualitative variables		Intervention		Control		Results of the *χ* ^2^ test
		Frequency	Percentage	Frequency	Percentage	
Gender	Male	12	40	14	46.7	*χ* ^2^ = 0.271 df = 1 *p*‐value = 0.602
	Female	18	60	16	53.3	
Marriage statue	Single	13	43.3	12	40	*χ* ^2^ = 1.040 df = 2 *p*‐value = 0.792
	Married	12	40	12	40	
	Divorced	5	16.7	6	20	
Education	Elementary	0	0	3	10.3	*χ* ^2^ = 12.64 df = 5 *p*‐value = 0.116
	High school	2	6.9	8	27.6	
	Diploma	11	37.9	12	41.4	
	Diploma associate	3	6.9	3	6.9	
	Bachelor's degree	12	41.4	4	13.8	
	Master's degree	2	6.9	0	0	
Occupation	Freelance	7	24.1	8	27.6	*χ* ^2^ = 5.75 df = 5 *p*‐value = 0.357
	Employee	9	31.0	3	10.3	
	Worker	0	0	2	6.9	
	Student	2	6.9	2	6.9	
	Housewife	5	17.2	8	27.6	
	Unemployed	6	20.7	6	20.7	
Income	Low	8	26.7	14	46.7	*χ* ^2^ = 2.60 df = 2 *p*‐value = 0.269
	Medium	20	66.7	14	46.7	
	High	2	6.6	2	6.6	
Disease history	Yes	6	20	11	36.7	*χ* ^2^ = 2.05 df = 1 *p*‐value = 0.152
	No	24	80	19	63.3	
Suicide history	Yes	10	33.3	14	46.7	*χ* ^2^ = 1.11 df = 1 *p*‐value = 0.292
	No	20	66.7	16	53.3	
Suicide time	Morning	5	16.7	7	23.3	*χ* ^2^ = 1.78 df = 2 *p*‐value = 0.411
	Evening	14	46.7	9	30	
	Night	11	36.7	14	46.7	
Suicide method	Taking poison	4	13.8	4	14.3	*χ* ^2^ = 6.24 df = 5 *p*‐value = 0.283
	Taking medication	19	65.5	16	57.1	
	Hanging	0	0	1	3.6	
	Self‐immolation	1	3.4	1	3.6	
	Falling	3	10.3	0	0	
	Cutting	2	6.9	6	21.4	
Causes of suicide	Incurable disease	1	3.4	0	0	*χ* ^2^ = 5.39 df = 5 *p*‐value = 0.422
	Depression	6	20.7	7	25.0	
	Divorce	6	20.7	2	7.1	
	Death of a loved one	0	0	2	7.1	
	Indebtedness	1	3.4	2	7.1	
	Other	15	51.7	15	53.6	
Age		Mean	SD	Mean	SD	Results of the independent‐samples t test
		27.7	7.23	29.26	9.07	*t* = 0.724 df = 58 *p*‐value = 0.472

### 3.2 Cognitive‐Behavioral Therapy

The results of the Kolmogorov–Smirnov test indicated that the scores for hopelessness and suicidal ideation were normally distributed across all measurement time points (*p* > 0.05).

In the intervention group, the mean scores of hopelessness at baseline, 1 month, and 3 months post‐intervention were 11.07 ± 2.31, 9.25 ± 1.22, and 8.03 ± 0.93, respectively. In contrast, the corresponding mean scores in the control group were 11.42 ± 2.46, 11.61 ± 1.69, and 11.00 ± 2.05, respectively (Table 3).

**Table 3:** Mean scores of despair in the control and intervention groups at before intervention,1 month after, and 3 months after the intervention.

Mean scores of despair		Frequency	Mean	SD
Before intervention	Intervention	30	11.071	2.319
	Control	30	11.42	2.46
A month after the intervention	Intervention	30	9.25	1.22
	Control	30	11.61	1.69
Three months after the intervention	Intervention	30	8.03	0.93
	Control	30	11.00	2.05

The results of the repeated measures ANOVA (rANOVA) revealed a statistically significant difference in the mean hopelessness scores between the intervention and control groups following the intervention (*p* < 0.05) (Table 4). Given that the interaction effect of time and intervention is significant, comparing the mean of despair between the intervention and control groups at different times is evaluated as the effect of the intervention. Bonferroni's multiple comparisons indicated that there was no statistically significant difference in mean hopelessness scores between the two groups before the intervention. (*p* = 0.598), indicating that the despair of the two groups was similar before the study. Also, a significant difference was observed in the difference in mean despair scores 1 month and 3 months after the intervention in the two groups (*p* < 0.001) (Table 5).

**Table 4:** Comparison of participants' despair scores between the two groups at three measurement time points based on the repeated measures ANOVA.

Mean scores of despair	RSS	df	MSE	F	*p* value	ηp2
The main effect of time	79.28	1	79.28	20.99	< 0.001	0.292
Group × time interaction effect	45.25	1	45.25	11.97	0.001	0.190
Error term (time)	192.65	51	3.77			
The main effect of the intervention	141.84	1	141.84	31.48	< 0.001	0.382
Error term (intervention)	229.75	51	4.50			

Abbreviations: ANOVA, analysis of variance; MSE, mean squared error; RSS, residual sum of squares; ηp2, partial eta squared.

**Table 5:** Mean despair score between intervention and control groups at measurement time points.

Timeline	Groups	Mean difference	Standard deviation difference	*p* value	Confidence interval	
					Lower bound	Upper bound
Before intervention	Intervention‐control	0.34	0.65	0.598	−0.97	1.66
One month after the intervention	Intervention‐control	2.35	0.40	< 0.001	1.54	3.17
Three months after the intervention	Intervention‐control	2.96	0.43	< 0.001	−3.84	−2.08

**Figure 2:**
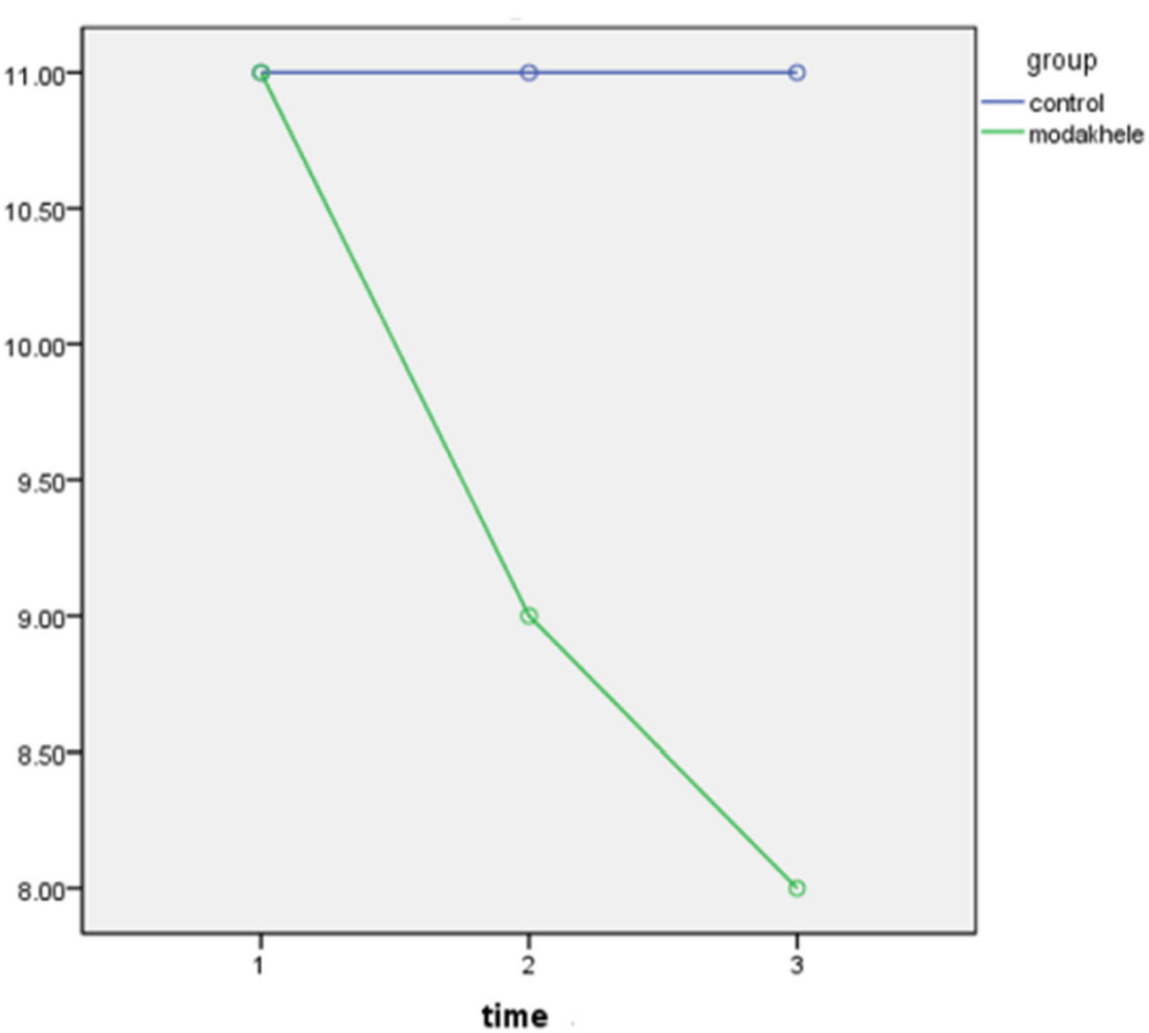
The trend of the mean score of suicidal ideation in three time points between the intervention and control groups.

In the intervention group, pairwise comparisons using the Bonferroni test revealed that the mean hopelessness scores at both post‐intervention time points (1 month and 3 months) were significantly lower than the baseline score (*p* < 0.05). Additionally, a significant difference was observed between the two post‐intervention scores (*p* = 0.011). In contrast, in the control group, no significant differences were found between the baseline and post‐intervention scores (*p* = 1.000), nor between the two post‐intervention time points (*p* = 0.41) (Figure 2; Table 6).

**Table 6:** Binary comparisons of participants' despair scores at three measurement time points based on the Bonferroni.

Groups	Time	Mean difference	Standard deviation difference	*p* value	Confidence interval	
					Lower bound	Upper bound
Control	Before and 1 month after the intervention	−0.19	0.47	1.000	−1.37	0.98
	Before and 3 months after the intervention	0.42	0.53	1.000	−0.91	1.75
	1 month and 3 months after the intervention	0.61	0.40	0.413	−0.39	1.62
Intervention	Before and 1 month after the intervention	1.81	0.46	0.001	0.65	2.97
	Before and 3 months after the intervention	3.03	0.52	< 0.001	1.72	4.34
	1 month and 3 months after the intervention	1.22	0.40	0.011	0.23	2.21

In the intervention group, the mean scores of suicidal ideation at baseline, 1 month, and 3 months post‐intervention were 21.14 ± 6.40, 6.14 ± 4.49, and 3.33 ± 2.74, respectively. In contrast, the corresponding scores in the control group were 23.73 ± 5.77, 23.80 ± 6.74, and 24.38 ± 5.80, respectively (Table 7).

**Table 7:** Mean suicidal ideation score of study participants in control and intervention groups.

Mean scores of suicidal ideations		Frequency	Mean	SD
Before intervention	Intervention	30	21.14	6.40
	Control	30	23.73	5.77
A month after the intervention	Intervention	30	6.14	4.49
	Control	30	23.80	6.74
Three months after the intervention	Intervention	30	3.33	2.74
	Control	30	24.38	5.80

The results of repeated measures ANOVA (rANOVA) indicated a statistically significant difference in mean suicidal ideation scores between the intervention and control groups following the intervention (*p* < 0.001) (Table 8). Bonferroni's multiple comparisons further revealed no significant difference in mean suicidal ideation between the two groups at baseline (*p* = 0.13), indicating that the suicidal ideation of the two groups was similar before the study. Also, a significant difference was observed in the difference in mean suicidal ideation scores 1 month and 3 months after the intervention in the two groups (*p* < 0.001) (Table 9).

**Table 8:** Mean suicidal ideation score of study participants in control and intervention groups.

Mean scores of suicidal ideations	RSS	df	MSE	F	*p* value	ηp2
The main effect of time	1950.36	1	1950.36	115.52	< 0.001	0.69
Group × time interaction effect	2258.92	1	2258.92	133.80	< 0.001	0.72
Error term (time)	860.97	51	16.88		—	
The main effect of the intervention	7528.39	1	7528.39	126.93	< 0.001	0.71
Error term (intervention)	3024.71	51	59.30		—	

Abbreviations: ANOVA, analysis of variance; MSE, mean squared error; RSS, residual sum of squares; ηp2, partial eta squared.

**Table 9:** Mean suicidal ideation score between intervention and control groups at measurement time points.

Timeline	Groups	Mean difference	Standard deviation difference	*p* value	Confidence interval	
					Lower bound	Upper bound
Before intervention	Intervention‐control	2.58	1.67	0.130	−0.78	5.94
One month after the intervention	Intervention‐control	17.66	1.56	< 0.001	14.51	20.80
Three months after the intervention	Intervention‐control	21.05	1.24	< 0.001	18.56	23.54

**Figure 3:**
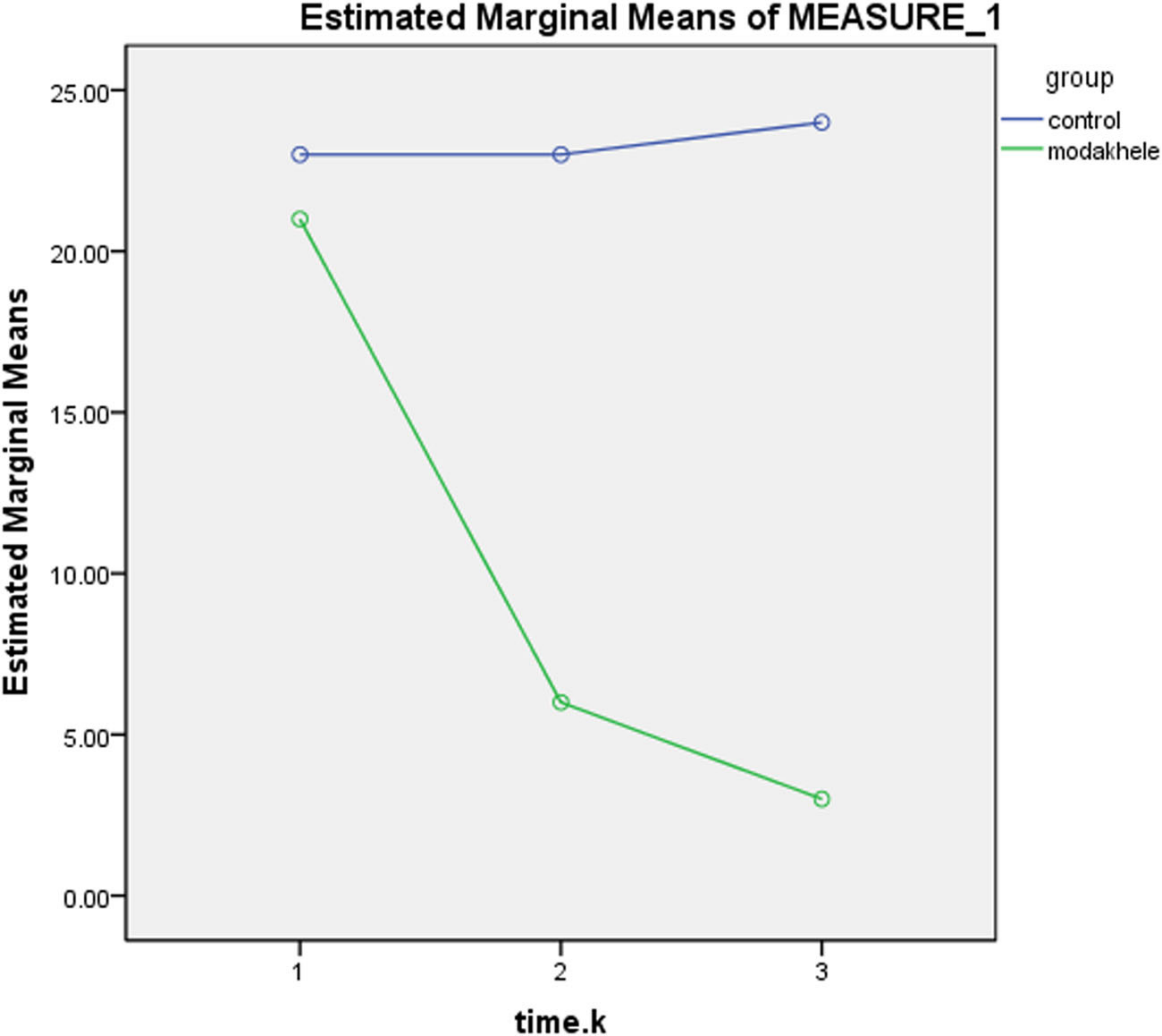
The trend of the mean score of despair in three time points between the intervention and control groups.

In the intervention group, pairwise comparisons using the Bonferroni test showed that the mean scores of suicidal ideation at both post‐intervention time points (1 month and 3 months) were significantly lower than the baseline score (*p* < 0.001). Additionally, a significant difference was observed between the two post‐intervention scores (*p* = 0.005). In contrast, in the control group, no significant differences were found between the baseline and post‐intervention scores (*p* = 1.000), nor between the two post‐intervention time points (*p* = 1.000). (Figure 3; Table 10).

**Table 10:** Binary comparisons of participants' suicidal ideation scores at three measurement time points based on the Bonferroni.

Groups	Time	Mean difference	Standard deviation difference	*p* value	Confidence interval	
					Lower bound	Upper bound
Control	Before and 1 month after the intervention	−0.077	1.23	1.000	−3.12	2.97
	Before and 3 months after the intervention	−0.65	1.14	1.000	−3.47	2.16
	1 month and 3 months after the intervention	−0.57	0.86	1.000	−2.71	1.55
Intervention	Before and 1 month after the intervention	15.00	1.20	< 0.001	12.00	17.99
	Before and 3 months after the intervention	17.81	1.11	< 0.001	15.04	20.58
	1 month and 3 months after the intervention	2.81	0.84	0.005	0.72	4.90

## 4 Discussion

The findings of this study showed that the two control and intervention groups did not have a statistically significant difference in terms of demographic variables that could affect the study results. Therefore, the existence of a substantial difference in dependent variables in the intervention groups before and after cognitive behavioral therapy was due to the positive effect of its implementation. Thus far, several studies have assessed the effect of cognitive behavioral therapy on suicidal patients. In contrast, the effect of cognitive behavioral therapy on suicidal ideation and despair among individuals who have attempted suicide has not been studied precisely.

The present study was conducted to determine the effect of cognitive behavioral therapy on the suicidal ideation of patients with attempted suicide. The findings of this study indicated a significant effect of time within the intervention group, as the mean score of suicidal ideation showed a statistically significant difference between pre‐ and post‐intervention assessments (*p* <0.05). Furthermore, the significant Group × Time interaction suggested that the mean scores of suicidal ideation differed significantly between the intervention and control groups across various time points. These findings suggest that the intervention had a beneficial effect on suicidal ideation. The statistically significant main effect of group indicates that, irrespective of the time points assessed, participants in the intervention group consistently exhibited lower mean scores of suicidal ideation compared to those in the control group.

The results of most studies in this area [33, 34, 35] are consistent with the results of our study, as we found that cognitive behavioral therapy can also be effective in patients with attempted suicide. Zemestani et al. (2015), in a study on the effectiveness of group cognitive‐behavioral therapy combined with family education on suicide‐related components in girls attempting suicide, indicated that there is a substantial reduction in depressive symptoms, suicidal ideation, and negative cognitive emotion regulation strategies, highlighting the positive impact of cognitive‐behavioral group therapy when integrated with family education [36]. Abbaspour et al. (2014) also showed that family‐based cognitive‐behavioral therapy and solution‐focused therapy have a positive effect on suicidal ideation, depression symptoms, family cohesion, and adaptability among individuals with a history of suicide attempts. The researchers concluded that both treatments achieved treatment goals, with family‐centered cognitive‐behavioral therapy exhibiting superior efficacy [37]. The findings of the aforementioned studies are consistent with the results of the present study.

Wenzel & Jager‐Hyman (2012) also indicated that direct treatment of suicidal‐related cognitions is needed, rather than treating depression to reduce suicidal ideation indirectly [38]. Tarrier et al. (2008), in a systematic review and meta‐analysis, concluded that interventions are effective only when they directly target specific components of suicidal behavior. However, treatments aimed at alleviating other symptoms, such as depression or psychological distress, to indirectly reduce suicidal behavior, were not found to be effective. Therefore, to achieve effectiveness, CBT‐based suicide prevention programs must be specifically designed, customized, and implemented to directly address suicidal behavior [39]. This treatment protocol was originally developed for patients with recent suicide attempts but can also be extended to individuals experiencing acute suicidal ideation that necessitates intervention [38]. In addition to targeting suicide‐related cognitions, cognitive therapists also focus on strengthening patients' problem‐solving abilities, conceptualizing suicidal behavior as a manifestation of ineffective problem‐solving [40]. Cognitive therapists systematically implement structured problem‐solving steps commonly found in cognitive‐behavioral protocols, including problem definition, generation of alternative solutions, decision‐making, and the implementation and evaluation of selected solutions [41]. Cognitive therapy has been shown to reduce the recurrence of suicidal behavior in adult suicide attempters compared to standard care. The key responsibility of cognitive behavioral therapists within clinical settings is to disseminate this approach, ensuring that individuals receive targeted, evidence‐based care following a suicidal crisis [38].

This study's findings also showed a statistically significant difference in the mean score of despair in the intervention group before, 1 month, and 3 months after the intervention. No meaningful difference was detected in the control group based on statistical analysis. Brown et al. (2006) also indicated that suicidal individuals exhibit higher levels of hopelessness [42].

A systematic review and meta‐analysis study by Tarrier et al. (2008) revealed that all measures of suicidal behavior showed significant reductions with treatment. However, in the two studies where hopelessness was the closest measure, this result did not show a significant reduction. In contrast, when all 10 studies that included the hopelessness measure were analyzed, a very significant reduction was observed in this measure. Thus, there appears to be a reduction across the spectrum of suicidal behavior with CBT treatment. As previously stated, we are very aware that measures of suicidal behavior are measures of death by suicide and cannot draw firm conclusions that CBT reduces actual suicide. Recognizing this, there is reasonable but cautious optimism that reductions in hopelessness and suicidal thoughts and behavior will result in a number, albeit unknown, of deaths by suicide prevented [39].

According to Wenzel and Jager‐Hyman (2012), in cognitive theory, suicidal risk increases with increasing severity of a single and comorbid psychiatric diagnosis because these clinical manifestations are likely to activate suicidality‐related schemas (e.g., hopelessness, unbearableness) [38]. Overall, the results of these studies support the effectiveness of cognitive behavioral therapy (CBT), a term used to describe cognitive therapy, for reducing suicidal thoughts, suicidal behavior, and hopelessness compared to usual care or treatment as usual (but not when compared to another active treatment) [38]. In addition, compared with patients who received usual care or treatment as usual (but not when compared to another active treatment), patients who received cognitive therapy scored lower on the Beck Hopelessness Scale (Beck & Steer, 1988) at 6‐month follow‐up [38]. The results of the above studies were consistent with the results of the present study.

### 4.1 Study Limitations

The COVID‐19 pandemic negatively impacted the study process, with many participants concerned about contracting the coronavirus. Participants received the intervention in small groups and were encouraged to follow all health protocols during the study period to prevent the spread of COVID‐19. One limitation of this study, which could limit the generalizability of the findings to larger populations, is the use of convenience sampling. It is recommended that future studies use random sampling with larger samples to increase the generalizability of the results.

Another limitation of the present study was that Participants in both study groups received treatment as usual (Psychiatric outpatient visits for treatment and counseling), but unfortunately, there was no facility to receive routine care from community physicians, as well as follow‐up and referral services from case managers in this study. Therefore, we had limitations regarding the possibility of group comparison regarding usual care.

## 5 Conclusion

In conclusion, the present results support the safety and efficacy of cognitive‐behavioral therapy for reducing despair and suicidal ideation in individuals who have attempted suicide. It can be said that cognitive behavioral therapy can increase therapeutic effectiveness due to its underlying mechanisms, such as acceptance, increased awareness, presence at the moment, and non‐judgmental observation. Future research is needed to evaluate efficacy, effectiveness, and cost‐effectiveness further. The task of cognitive behavioral therapists in the organization is to disseminate this therapy so that people with suicidal tendencies can receive the targeted care they need after a course of treatment.

## Author Contributions

**Zahra Kermanshahi:** investigation, writing – original draft, writing – review and editing, resources. **Leyla Alilu:** conceptualization, writing – review and editing, methodology, supervision, writing – original draft, investigation, project administration. **Moulad Radfar:** conceptualization. **Behzad Bushehri:** investigation. **Hamidreza Khalkhali:** software, formal analysis.

## Conflicts of Interest

The authors affirm the absence of any financial conflicts or personal affiliations that might be perceived as influencing this study.

## 1 Transparency Statement

As the lead researcher, Leyla Alilu certifies that this manuscript presents a truthful, precise, and complete representation of the reported study. All significant aspects have been included, and any deviations from the original study design (including protocol registration, where applicable) have been properly documented and justified.

## Data Availability

All contributing authors have reviewed and endorsed the final manuscript. The corresponding author assumes full responsibility for both data integrity and analytical accuracy, having had unrestricted access to all study data. The data that support the findings of this study are available on request from the corresponding author. The data are not publicly available due to privacy or ethical restrictions.
